# Risk Stratification for In-Hospital Mortality in Alzheimer’s Disease Using Interpretable Regression and Explainable AI

**DOI:** 10.3390/geriatrics11020023

**Published:** 2026-02-24

**Authors:** Tursun Alkam, Ebrahim Tarshizi, Andrew H. Van Benschoten

**Affiliations:** Master’s Program of Applied Artificial Intelligence, University of San Diego, San Diego, CA 92110, USA

**Keywords:** Alzheimer’s disease, in-hospital mortality, XGBoost, SHAP, logistic regression, risk prediction, comorbidities, explainable AI

## Abstract

Background: Older adults with Alzheimer’s disease (AD) face a heightened risk of adverse hospital outcomes, including mortality. However, early identification of high-risk patients remains a challenge. While regression models provide interpretable associations, they may miss non-linear interactions that machine learning can uncover. Objective: To identify key predictors of in-hospital mortality among AD patients using both survey-weighted logistic regression and explainable machine learning. Methods: We analyzed hospitalizations among AD patients aged ≥60 in the 2017 Nationwide Inpatient Sample (NIS). The outcome was in-hospital death. Predictors included demographics, hospital variables, and 15 comorbidities. Logistic regression used survey weighting to generate nationally representative inference; XGBoost incorporated NIS discharge weights as sample weights during 5-fold hospital-grouped cross-validation and used the same weights in performance evaluation. Missing-value imputation and feature scaling were performed within the cross-validation pipelines to prevent data leakage. Model performance was assessed using AUROC, AUPRC, Brier score, and log loss. Feature importance was assessed using adjusted odds ratios and SHapley Additive exPlanations (SHAP). A sensitivity analysis excluded palliative care and DNR status and was re-evaluated under the same grouped cross-validation. Results: In the full model, logistic regression achieved AUROC 0.879 and AUPRC 0.310, while XGBoost achieved AUROC 0.887 and AUPRC 0.324. Palliative care (aOR 6.19), acute respiratory failure (aOR 5.15), DNR status (aOR 2.20), and sepsis (aOR 2.26) were the strongest logistic predictors. SHAP analysis corroborated these findings and additionally emphasized dysphagia, malnutrition, and pressure ulcers. In sensitivity analysis excluding palliative care and DNR status, logistic regression performance declined (AUROC 0.806; AUPRC 0.206), while XGBoost performed similarly (AUROC 0.811; AUPRC 0.206). SHAP corroborated the dominant signals from end-of-life documentation and acute organ failure in the full model; in the restricted model (excluding DNR and palliative care), SHAP highlighted physiologic and frailty-related features (e.g., dysphagia, malnutrition, aspiration risk) that may be more actionable when end-of-life documentation is absent. Conclusions: Combining regression with explainable machine learning enables robust mortality risk stratification in hospitalized AD patients. Restricted models excluding end-of-life indicators provide actionable risk signals when such documentation is absent, while the full model may better support resource allocation and goals-of-care workflows.

## 1. Introduction

Alzheimer’s disease (AD) is a progressive neurodegenerative disorder affecting over 6 million individuals in the United States and contributing significantly to healthcare burden and mortality among older adults [1]. As cognitive decline advances, patients with AD become increasingly susceptible to acute medical complications, necessitating hospitalization [2,3,4]. In-hospital mortality among AD patients is a particularly serious outcome, yet its predictors remain incompletely understood and often under-recognized in routine care [3,4].

Prior research has examined general risk factors for in-hospital mortality among older adults, including age, comorbidity burden, and acute organ failure [4,5,6,7,8]. However, few studies have focused specifically on hospitalized patients with AD, a population whose vulnerabilities, including frailty, atypical presentations, and limited physiological reserve, may alter risk profiles and challenge traditional prognostic models [9]. Additionally, much of the existing literature has relied exclusively on regression-based approaches, which, while valuable for inferential clarity, may underemphasize complex interactions or system-level factors relevant to real-world clinical decision-making [9,10,11,12,13].

To address these limitations, we employed a dual-analytic strategy integrating traditional multivariable logistic regression with explainable artificial intelligence (AI) using SHapley Additive exPlanations (SHAP) applied to an eXtreme Gradient Boosting (XGBoost) classifier [14,15]. This approach allows for both statistical inference (via odds ratios) and nuanced model interpretability (via SHAP importance rankings), facilitating identification of both terminal and potentially modifiable predictors. We used the 2017 National Inpatient Sample (NIS), a large, nationally representative dataset of U.S. hospitalizations, to assess predictors of in-hospital mortality among older adults with AD. To evaluate the robustness of our findings, we also conducted a sensitivity analysis.

This work contributes to the growing field of interpretable AI in clinical epidemiology and aims to inform risk stratification and early intervention strategies for hospitalized patients with AD.

## 2. Methods

### 2.1. Data Source and Study Population

This study used data from the 2017 NIS, a component of the Healthcare Cost and Utilization Project (HCUP) developed by the Agency for Healthcare Research and Quality (https://www.hcup-us.ahrq.gov/nisoverview.jsp, accessed on 1 January 2026). The NIS is the largest publicly available all-payer inpatient database in the United States, representing approximately 35 million weighted hospitalizations annually from over 4500 hospitals across 47 states. It includes comprehensive patient- and hospital-level variables, such as diagnoses, procedures, demographics, and discharge outcomes.

The NIS is a discharge-level database in which each record represents a single hospitalization rather than a unique patient. Because patient identifiers are not available, the NIS does not permit longitudinal linkage across admissions; therefore, repeat hospitalizations by the same individual cannot be identified or removed.

We included hospitalizations for patients aged 60 years or older with a diagnosis of AD, defined using ICD-10-CM codes G30.0, G30.1, G30.8, and G30.9. AD status was identified from any of the 40 available diagnosis fields (I10_DX1-I10_DX40).

### 2.2. Identification of Predictors and Variable Construction

To construct clinically relevant features for modeling, we first tabulated the most frequent ICD-10-CM diagnosis codes (Supplementary Table S1) among AD patients who died during hospitalization. These included acute conditions (e.g., sepsis, acute respiratory failure, acute kidney injury, aspiration pneumonia), chronic diseases (e.g., congestive heart failure, coronary artery disease, cerebrovascular disease, atrial fibrillation, anemia, hypothyroidism), and indicators of functional and nutritional decline (e.g., urinary tract infection, dysphagia, pressure ulcers, malnutrition).

We also included two administrative variables, do-not-resuscitate (DNR) orders and receipt of palliative care services, which, although reflective of end-of-life decision-making, were retained due to their clinical relevance and high prevalence in this population. For sensitivity analysis, these two variables were excluded in a secondary modeling pipeline to evaluate their effect on model performance and feature rankings.

### 2.3. Covariates and Feature Engineering

In addition to clinical predictors, we included sociodemographic and hospital-level variables previously associated with mortality. Patient-level covariates included age (continuous), sex (binary), race/ethnicity (categorized into five standard groups), ZIP-code-based income quartile (1 = lowest, 4 = highest), weekend admission (yes/no), elective versus emergency admission, and inter-facility transfer status. Hospital-level characteristics included U.S. Census division (nine categories), used as a proxy for geographic variation in practice patterns. All variables were harmonized into interpretable binary or categorical indicators as appropriate.

All preprocessing steps were implemented within scikit-learn pipelines to prevent data leakage. Missing values were imputed using SimpleImputer (median for continuous variables; constant 0 for binary indicators; most-frequent for categorical variables). Continuous and binary predictors were standardized using StandardScaler. All transformations were fit on each training fold and applied to the corresponding held-out fold within the grouped cross-validation pipeline. Missing data were modest and primarily limited to selected sociodemographic variables. Imputation was performed within each training fold (median for continuous variables; most-frequent for categorical variables; and constant 0 for binary indicators) and applied to the corresponding held-out fold within the grouped cross-validation pipeline. Survey discharge weights were applied during model fitting and performance evaluation (including cross-validation), consistent with the NIS sampling design.

### 2.4. Descriptive Statistics and Logistic Regression Analysis

Survey-weighted descriptive statistics were generated using Stata 16.0. Categorical variables were summarized as proportions with 95% confidence intervals (CIs), and continuous variables were reported as means with 95% CIs. Discharge-level weights provided by HCUP were applied to produce nationally representative estimates.

Multivariable logistic regression was used to identify adjusted risk factors for in-hospital mortality. All selected clinical, demographic, and hospital variables were entered into the model simultaneously. Adjusted odds ratios (aORs) with 95% CIs were reported, and statistical significance was determined using Wald tests with a two-sided *p*-value threshold of <0.05. Because the NIS is a complex survey dataset, regression inference relied on design-based Wald tests, which are standard for survey-weighted models and provide hypothesis tests for coefficients analogous to *z*/*t* tests in unweighted regression.

To mitigate redundancy and potential collinearity among regression predictors, we reviewed pairwise correlations and clinical overlap among candidate variables, and performed a prespecified sensitivity analysis excluding end-of-life documentation variables (DNR status and palliative care), which are expected to be highly correlated with mortality.

Survey weighting was performed using the HCUP-provided NIS discharge weights (DISCWT) to generate nationally representative estimates, consistent with HCUP’s NIS methodology. All weighted descriptive statistics and survey-weighted regression models incorporated the NIS sampling design to account for stratification and clustering.

### 2.5. Machine Learning Modeling and Performance Evaluation

For predictive modeling, we implemented two supervised classifiers: logistic regression (an interpretable baseline) and XGBoost, an ensemble method capable of capturing non-linear effects in structured administrative and clinical data. Models were evaluated using 5-fold grouped cross-validation (GroupKFold) clustered by hospital identifier, and HCUP discharge weights were incorporated as sample weights. All preprocessing (imputation and scaling) was performed within each training fold and applied to the corresponding held-out fold.

XGBoost hyperparameters were prespecified a priori rather than optimized using automated hyperparameter search.

We prespecified XGBoost hyperparameters a priori rather than performing automated tuning (e.g., random search or Bayesian optimization) because (i) extensive tuning can inflate model-development degrees of freedom and inadvertently overfit to dataset-specific idiosyncrasies even under cross-validation, particularly with rare outcomes and correlated predictors; (ii) iterative search is computationally intensive under 5-fold hospital-grouped cross-validation; and (iii) prespecification improves transparency and facilitates replication and external validation in subsequent NIS cohorts. Accordingly, we used a conservative configuration commonly recommended for clinical tabular data to prioritize stability and interpretability over marginal performance gains. Specifically, we set n_estimators = 600, learning_rate = 0.05, max_depth = 4, subsample = 0.9, colsample_bytree = 0.9, and reg_lambda = 1.0, combining shrinkage, limited depth, row/column subsampling, and L2 regularization to reduce overfitting. Future work may evaluate systematic tuning within a nested hospital-grouped cross-validation framework.

Because mortality is imbalanced, we report AUROC and AUPRC alongside calibration metrics. We did not apply synthetic over/undersampling or XGBoost class reweighting in the primary analysis. Calibration was assessed using the Brier score (mean squared error of predicted probabilities) and log loss (which penalizes overconfident incorrect predictions), where lower values indicate better probabilistic calibration.

### 2.6. Sensitivity Analysis

To assess whether end-of-life documentation might dominate predictive signals, we conducted a sensitivity analysis excluding palliative care and DNR variables from both the logistic regression and XGBoost models. Restricted-model performance was re-evaluated using the same hospital-grouped cross-validation and preprocessing pipelines, and SHAP values were recalculated for the restricted feature set to assess changes in feature importance rankings. For the restricted analysis, SHAP values were recomputed from the restricted XGBoost model to assess whether feature importance patterns shifted after exclusion of end-of-life indicators.

### 2.7. Model Explainability Using SHAP Values

To enhance interpretability of the XGBoost model, we utilized SHAP, a game-theoretic approach that assigns each feature an importance value for a particular prediction [14]. Specifically, we employed the TreeExplainer algorithm, which provides exact computation of Shapley values for tree-based ensemble models and enables the visualization of non-linear feature effects and interactions [16].

We calculated SHAP values on a random subsample of 5000 observations from the full dataset to balance interpretability and computational efficiency. Two visualization types were generated: (1) a SHAP summary plot illustrating the direction and distribution of feature effects across all patients, and (2) a SHAP bar plot ranking features by their mean absolute SHAP value, indicating average influence on model output magnitude. While SHAP provides additive explanations for model behavior, interpretations can be sensitive to correlated predictors and should be viewed as explanatory rather than causal.

### 2.8. Software and Reproducibility

All data cleaning, modeling, and visualization were conducted using Python 3.11 in Google Colab. Key libraries included pandas (v1.5), scikit-learn (v1.4), xgboost (v2.0.0), shap (v0.45.0), and matplotlib (v3.7). Initial variable construction, descriptive analysis, and survey weighting were performed in Stata 16.0. All source code, annotated notebooks, and documentation are publicly available on GitHub at https://github.com/TAlkam/NIS (accessed on 1 January 2026).

## 3. Results

### 3.1. Patient Characteristics

Table 1 summarizes the demographic and clinical characteristics of hospitalized patients with AD, aged 60 and older, in the 2017 NIS cohort. The weighted sample included 88,875 AD-related hospitalizations, of which 4.7% resulted in in-hospital mortality. The mean age was 82.4 years (95% CI: 82.33–82.47), and 61.7% were female. Most admissions were non-elective (92.4%), with 24.2% occurring on weekends. Approximately 17.5% of patients were transferred from other facilities, and admissions were distributed across all nine hospital census divisions. Socioeconomic distribution was relatively even, with 28.9% of patients residing in the lowest ZIP income quartile.

The most prevalent clinical diagnoses included urinary tract infection (25.4%), coronary artery disease (25.7%), atrial fibrillation (25.6%), acute kidney injury (23.2%), and congestive heart failure (23.0%). Hypothyroidism (21.7%), pressure ulcers (7.2%), malnutrition (8.2%), and dysphagia (10.7%) were also frequent. Notably, 32.1% of patients had documented do-not-resuscitate (DNR) orders, and 11.3% received palliative care services during the hospitalization.

### 3.2. Risk Factors Identified via Logistic Regression

Multivariable survey-weighted logistic regression results are presented in Table 2. Several acute complications and end-of-life care variables emerged as the strongest predictors of in-hospital mortality. Receipt of palliative care was associated with a markedly elevated risk (adjusted odds ratio [aOR] 6.19; 95% CI: 5.59–6.85), followed by acute respiratory failure (aOR 5.15), DNR status (aOR 2.20), and sepsis (aOR 2.26). Other significant contributors included acute kidney injury, aspiration pneumonia, malnutrition, cerebrovascular disease, and atrial fibrillation. DNR status and receipt of palliative care should be interpreted primarily as markers of clinician-recognized illness severity and end-of-life trajectory and associated care decisions, rather than as causal determinants of mortality risk. Accordingly, these variables should not be interpreted as evidence that palliative care increases the risk of death.

Interestingly, some variables showed protective or inverse associations with mortality. Dysphagia was associated with lower odds of death (aOR 0.57), possibly reflecting early intervention or feeding-related vigilance. Similarly, anemia, higher ZIP income quartiles, and certain hospital divisions were associated with lower mortality. Elective admissions (aOR 2.33) and transfer-in status (aORs ranging from 1.12 to 1.56) were associated with elevated risk, likely reflecting patient acuity and care complexity.

### 3.3. Model Performance Metrics

To support clinical interpretability across different phases of hospitalization, we report results for two complementary models: a full model including end-of-life documentation variables (DNR status and palliative care) and a restricted model excluding these variables. The restricted model is intended to reflect admission-level risk assessment before end-of-life documentation is available and to emphasize more actionable physiologic and care-pathway predictors, whereas the full model better reflects recognized end-of-life trajectories relevant to care planning and resource allocation.

As shown in Table 3, XGBoost demonstrated slightly higher discrimination than logistic regression and modestly better calibration. The XGBoost model achieved an AUROC of 0.887 and an AUPRC of 0.324, compared to 0.879 and 0.310, respectively, for logistic regression. Calibration metrics also favored XGBoost, with a lower Brier score (0.036 vs. 0.037) and log loss (0.134 vs. 0.138). Given the imbalanced outcome (4.7% mortality), AUPRC is reported alongside AUROC to better reflect minority-class detection performance.

### 3.4. Top Predictors: SHAP vs. Regression

**Figure 1:**
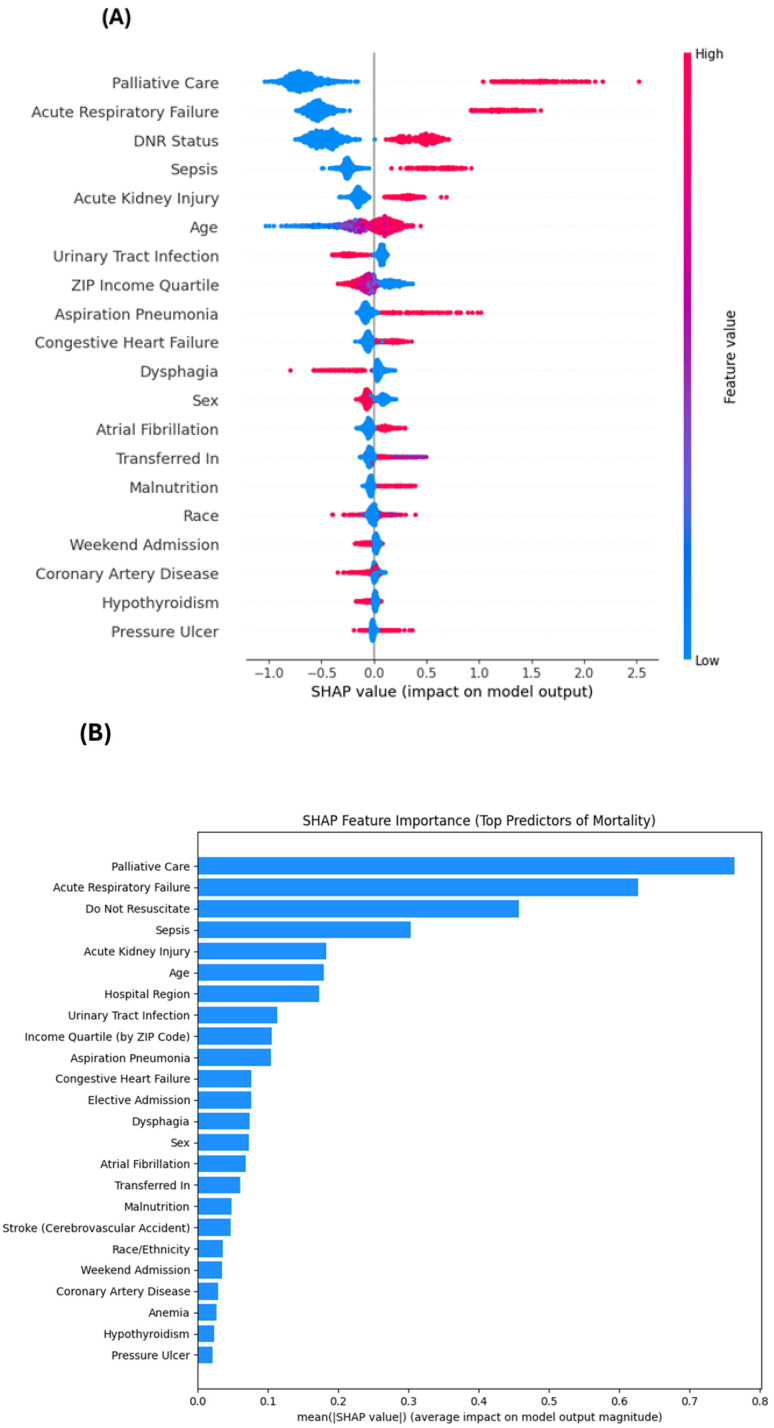
SHAP interpretation of the full-model XGBoost predictor for in-hospital mortality among hospitalized patients with AD. (**A**) SHAP summary (beeswarm) plot showing the top features influencing the full-model XGBoost prediction of in-hospital mortality. Each dot represents an individual admission; the x-axis shows the SHAP value (direction and magnitude of a feature’s contribution to predicted mortality risk). Features are ranked by mean absolute SHAP value (global importance). Dot color indicates the original feature value (red = higher, blue = lower). Prominent predictors include palliative care, acute respiratory failure, Do-Not-Resuscitate (DNR) status, sepsis, acute kidney injury, and age, with additional clinical factors (e.g., dysphagia, aspiration pneumonia, malnutrition) contributing to model predictions. Positive SHAP values indicate increased predicted mortality risk; negative values indicate decreased predicted risk. (**B**) Global SHAP feature importance (bar) plot for the same full model. Importance is quantified as the mean absolute SHAP value, representing each variable’s average contribution to the magnitude of the model output (independent of direction). Predictors are displayed in descending order, highlighting major signals from acute physiological conditions and care-status variables. SHAP values were computed using a tree-based SHAP explainer optimized for XGBoost.

Table 4 presents the top features ranked by XGBoost gain and logistic regression coefficients. Palliative care consistently emerged as the most influential variable across both models. Acute respiratory failure, sepsis, DNR orders, and acute kidney injury followed closely, highlighting the predominance of acute physiological insults in driving mortality. While survey-weighted regression (Table 2) showed inverse associations for dysphagia and anemia, predictive logistic coefficients (Table 4) may differ in direction for correlated predictors and should be interpreted as model-specific predictive weights, not causal effects. A notable divergence was observed for dysphagia: while it appeared protective in the multivariable survey-weighted logistic model (aOR 0.569, 95% CI: 0.50–0.64), it ranked among the top global predictors in the XGBoost model. This discrepancy suggests that swallowing dysfunction may confer risk primarily through complex, context-dependent interactions with co-occurring frailty and related complications (e.g., aspiration risk, malnutrition, infection burden), rather than acting as a simple independent linear predictor. In the SHAP analysis (Figure 1), these variables can contribute to higher predicted mortality risk in specific patient contexts, consistent with non-linear interactions and comorbidity patterns that are not captured by additive regression terms. The SHAP bar plot further indicated that palliative care, acute respiratory failure, DNR status, sepsis, acute kidney injury, and age accounted for the majority of global model influence.

### 3.5. Explainable Machine Learning Interpretation

Figure 1A displays the SHAP summary plot, illustrating how feature values affect individual predictions in the full model. Higher feature values for palliative care, acute respiratory failure, and DNR status were associated with positive SHAP values, consistent with increased predicted mortality risk. In contrast, several comorbidity and administrative variables showed more heterogeneous SHAP distributions, indicating context-dependent effects likely driven by interactions and correlated clinical profiles. Figure 1B visualizes mean absolute SHAP values across features, reinforcing that end-of-life documentation and acute organ failures were the most consistently impactful contributors, while administrative and socioeconomic factors remained non-negligible. For readers less familiar with SHAP: in the summary (beeswarm) plot, each dot represents an individual hospitalization; dots positioned to the right indicate increased predicted mortality risk, while dots to the left indicate decreased risk, and dot color reflects higher (red) or lower (blue) feature values.

### 3.6. Sensitivity Analysis Excluding End-of-Life Predictors

**Figure 2:**
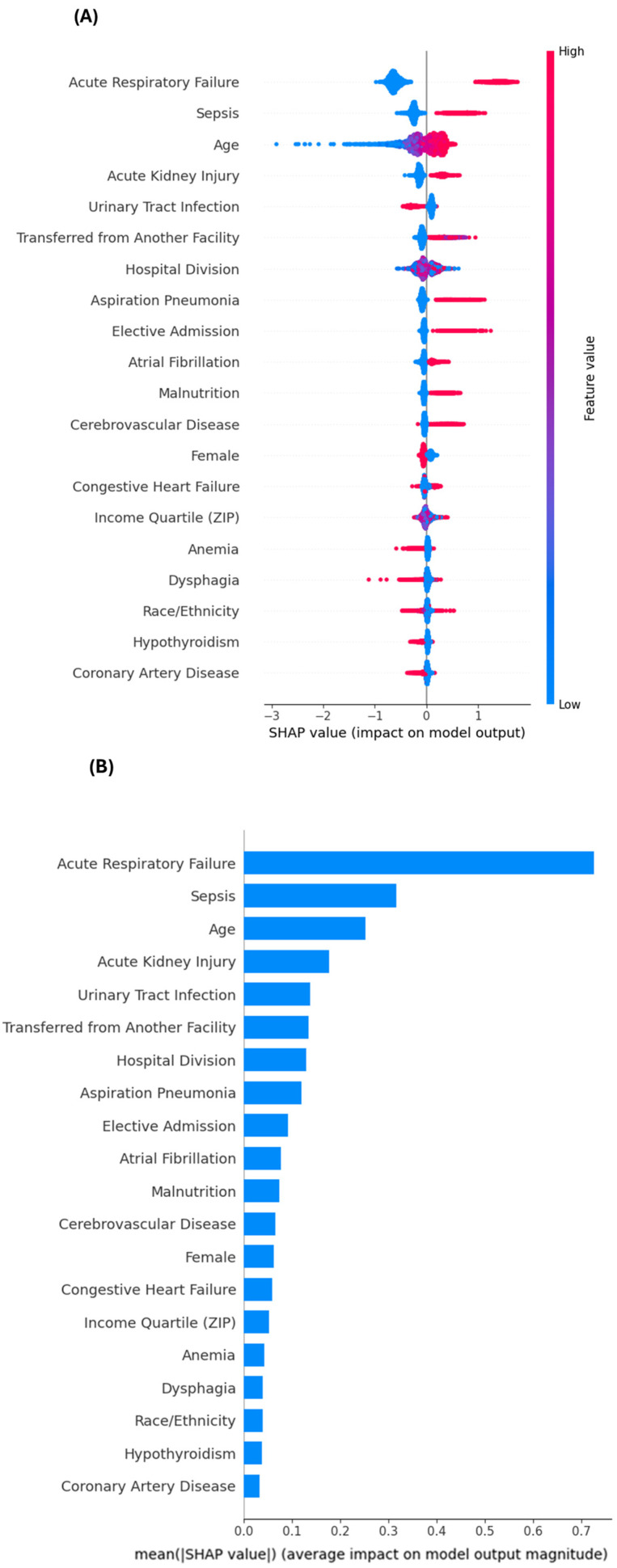
Restricted-model SHAP interpretation for in-hospital mortality prediction among hospitalized patients with AD (excluding end-of-life indicators). (**A**) SHAP summary (beeswarm) plot showing the top features influencing the restricted XGBoost model after excluding Do-Not-Resuscitate (DNR) status and palliative care. Each dot represents an individual admission; the x-axis shows the SHAP value (direction and magnitude of a feature’s contribution to predicted mortality risk). Features are ranked by mean absolute SHAP value (global importance). Dot color indicates the original feature value (red = higher, blue = lower). Positive SHAP values indicate increased predicted mortality risk; negative values indicate decreased predicted risk. (**B**) Restricted-model global SHAP feature importance (bar) plot for the same model. Importance is quantified as the mean absolute SHAP value, representing each variable’s average contribution to the magnitude of the model output (independent of direction). Predictors are displayed in descending order, highlighting persistent physiologic and care-pathway signals even when end-of-life documentation is unavailable.

To assess robustness and reduce dependence on end-of-life documentation, we repeated model development after excluding palliative care and DNR status and re-evaluated both models using the same hospital-grouped cross-validation pipeline. Because these variables are strongly associated with mortality, they may mask other clinically actionable predictors. Under the restricted feature set, discrimination decreased and was similar across models (logistic regression: AUROC = 0.806; AUPRC = 0.206; Brier = 0.040; log loss = 0.157; XGBoost: AUROC = 0.811; AUPRC = 0.206; Brier = 0.040; log loss = 0.156). Restricted-model SHAP analyses (Figure 2A,B) showed that acute respiratory failure, sepsis, age, and acute kidney injury remained the dominant contributors to mortality risk, followed by urinary tract infection, transfer-in status, hospital division, aspiration pneumonia, and elective admission. Collectively, these findings indicate that clinically interpretable physiologic and care-trajectory signals persist even when end-of-life documentation is unavailable; accordingly, the restricted model may better support actionable risk stratification earlier in hospitalization, whereas the full model may better support resource allocation and goals-of-care workflows.

## 4. Discussion

In this nationally representative study of older adults hospitalized with AD, we employed both survey-weighted logistic regression and explainable machine learning to identify predictors of in-hospital mortality. The models developed in this study are intended to support clinical decision-making rather than replace clinician judgment. In practice, the restricted model excluding DNR orders and palliative care may be most useful early during hospitalization, when end-of-life documentation is not yet available, to support admission-level risk stratification and prompt enhanced monitoring and timely multidisciplinary interventions (e.g., early respiratory surveillance, nutritional assessment, and speech-language pathology evaluation when indicated). As hospitalization progresses, the full model incorporating DNR status and palliative care may better reflect recognized end-of-life trajectories and support goals-of-care discussions, care coordination, and resource allocation. These complementary use-cases illustrate how interpretable prediction tools could be operationalized across different stages of inpatient care in older adults with AD.

The mortality outcome in this study reflects in-hospital death during the index hospitalization and should not be interpreted as an estimate of annual or all-cause mortality in older adults with AD. Many deaths occur after discharge or in non-hospital settings, including long-term care facilities and hospice; therefore, our results should be interpreted as identifying predictors of short-term inpatient mortality risk within acute hospital care. This dual-analytical approach enabled us to confirm known risk factors, surface novel insights, and compare linear versus non-linear feature effects [4,17,18,19,20,21,22]. By integrating SHAP with XGBoost modeling, we offer a transparent, interpretable framework that augments traditional epidemiologic inference and supports clinical decision-making in complex, multimorbid populations [14,15,16,23,24,25,26,27].

### 4.1. Key Mortality Predictors: Traditional and Novel Contributors

Our regression model reaffirmed the central role of acute physiological insults, including sepsis, ARF, and AKI, as dominant drivers of mortality in hospitalized AD patients [28,29,30]. Age, male sex, lower ZIP income quartile, elective admission, and interfacility transfer also independently increased the odds of death. Additionally, markers of end-of-life care, particularly palliative consultations and DNR orders, were among the strongest correlates, likely reflecting terminal illness recognition and advanced care planning rather than modifiable risk factors [18,31,32].

The machine learning model, while confirming many of the same variables, revealed additional nuances. SHAP interpretation showed that some variables traditionally viewed as less critical, such as dysphagia and anemia, had consistently high feature importance. Dysphagia, for example, emerged as a prominent mortality predictor in XGBoost but showed a protective association in regression, highlighting potential non-linear or interaction effects. Dysphagia, malnutrition, and pressure ulcers are well-recognized markers of vulnerability in older adults and dementia. The contribution of our study is not to propose them as novel risk factors, but to quantify and rank their relative importance for in-hospital mortality in a large, nationally representative cohort using an explainable machine learning framework. This discrepancy points to the clinical complexity of AD patients, where certain conditions may shift from protective to harmful depending on comorbidity clusters or care trajectories [33,34,35,36]. The prominence of aspiration pneumonia and malnutrition in both modeling frameworks further supports the hypothesis that frailty and swallowing dysfunction represent under-recognized but modifiable mortality risks in late-stage AD [18,37,38,39].

SHAP values provide a model-based explanation of how features contribute to the predicted risk under the observed data distribution; they do not imply that changing a feature would causally change mortality risk. Because administrative predictors may be correlated and reflect shared pathways (e.g., illness severity, treatment intensity, or documentation practices), SHAP should be interpreted as an explanatory tool for model behavior rather than causal inference, which would require additional assumptions and study designs beyond the scope of the NIS.

### 4.2. Concordance and Divergence Between Modeling Approaches

Our comparative analysis reveals areas of agreement and divergence between regression and machine learning approaches. Strong concordance was observed for major acute conditions (e.g., ARF, sepsis, AKI) and for administrative markers of end-of-life care (e.g., palliative consults, DNR orders). However, several variables with limited significance in the regression model, including hypothyroidism, anemia, and race, showed greater relative importance in SHAP plots. These differences may reflect the inability of regression models to fully capture complex, non-linear interactions or latent effects from feature combinations [40]. Conversely, features such as pressure ulcers, hypothesized to be predictive based on prior literature, ranked lower in both models, suggesting that while clinically salient, they may not independently drive mortality in this population [41]. Although XGBoost demonstrated slightly higher discrimination and modestly better calibration than survey-weighted logistic regression, the performance gains were small. In clinical settings where transparency, simplicity, and ease of implementation are prioritized, survey-weighted regression may be preferable, while explainable machine learning may add value by revealing non-linear and interaction-driven risk patterns. This divergence underscores the value of methodological pluralism. Rather than framing machine learning as a replacement for regression, our study highlights its utility as a complementary tool, particularly in contexts like dementia care where heterogeneity in disease progression, coding variability, and comorbidity patterns challenge single-model inference.

Despite only marginal improvements in aggregate discrimination metrics, tree-based models may still be advantageous in settings where risk is shaped by non-linearities and interactions that are difficult to prespecify in regression (e.g., threshold effects or combinations of acute illness markers and frailty). In this study, XGBoost added value by surfacing interaction-driven signals and non-linear contributions of less intuitive features (e.g., dysphagia and anemia) while retaining interpretability through SHAP. Accordingly, we view explainable tree-based models as particularly useful for exploratory analyses, hypothesis generation about interaction effects, and early risk stratification workflows, while acknowledging that regression may remain preferable when maximum simplicity, transparency, and ease of implementation are prioritized.

### 4.3. Interpretation of Apparently Paradoxical Predictors

Several predictors showed seemingly paradoxical patterns, including dysphagia, anemia, and ICU admission, which were associated with lower odds of in-hospital mortality in survey-weighted regression yet ranked among influential features in the XGBoost/SHAP analysis. These discrepancies likely reflect care trajectories and clinical context rather than true protective physiology. For example, documentation of dysphagia may prompt increased vigilance and interventions (e.g., aspiration precautions, diet modification, early speech-language pathology consultation), which can mitigate downstream complications. Similarly, anemia and ICU admission may function as markers of treatment intensity, monitoring, and triage pathways, and may be differentially coded across hospitals. In addition, non-linear interactions between these variables and frailty, comorbidity burden, and acute illness severity may be captured by machine learning models but not by additive regression terms. Collectively, these findings reinforce that some administrative predictors should be interpreted as indicators embedded within broader care processes rather than isolated biological risk factors.

### 4.4. Insights from Sensitivity Analyses

We conducted a sensitivity analysis excluding both palliative care and DNR status to evaluate whether end-of-life documentation dominated predictive performance. Under this restricted feature set, both models exhibited lower discrimination and calibration and performed similarly, consistent with the interpretation that these end-of-life variables capture substantial prognostic information in administrative data.

Notably, several clinical drivers including acute respiratory failure, sepsis, acute kidney injury, and age remained influential after exclusion of end-of-life indicators. In restricted-model SHAP rankings (Figure 2), urinary tract infection, transfer-in status, hospital division, aspiration pneumonia, and elective admission also emerged among the leading contributors. Dysphagia remained among the top features in restricted-model SHAP rankings, supporting its role as a marker of frailty, aspiration risk, or impaired feeding mechanisms; however, such signals should be interpreted in the context of potential medical coding variation and correlated comorbidity patterns.

Clinically, this distinction supports two complementary use-cases: the restricted model is better aligned with actionable risk stratification in admissions without explicit end-of-life documentation (supporting earlier intervention and care planning), whereas the full model may be more useful for resource allocation and goals-of-care workflows where palliative care and DNR appropriately reflect recognized terminal trajectories.

### 4.5. Clinical Applicability of the Risk Stratification Models

The models developed in this study are intended to support clinical decision-making rather than replace clinician judgment. In practice, the restricted model excluding DNR orders and palliative care is likely most useful early in the hospitalization, when advance care planning documentation may be incomplete or not yet recorded. An admission-level risk estimate could be operationalized as a decision-support signal to identify hospitalized older adults with Alzheimer’s disease who may benefit from early escalation of monitoring and timely multidisciplinary assessment. For example, patients flagged as high-risk could receive closer respiratory surveillance (given the prominence of acute respiratory failure and sepsis), earlier evaluation for infection and organ dysfunction, and proactive prevention strategies (e.g., aspiration precautions when dysphagia risk is suspected, medication review, early mobilization when feasible). Similarly, a high-risk designation could prompt early screening for malnutrition and dehydration, expedited dietitian involvement, and targeted consultation for speech-language pathology when swallowing impairment is documented or clinically suspected. Importantly, the restricted model is not intended to dictate care but to prioritize attention and resources when clinical teams face competing demands and limited capacity.

As hospitalization progresses, the full model incorporating DNR status and palliative care may better reflect recognized end-of-life trajectories and evolving goals of care. In this setting, predicted risk may be most helpful for supporting structured communication rather than acute triage. For example, alignment of risk estimates with clinician assessment can facilitate earlier family engagement, clarify prognosis, and support shared decision-making regarding treatment intensity, comfort-focused care, discharge planning, and transitions to hospice or post-acute services when appropriate. Because DNR orders and palliative care are best interpreted as markers of clinician-recognized severity and care decisions, their predictive contribution should not be construed as causal. Instead, the value of the full model lies in characterizing how real-world care pathways and documented severity signals align with short-term inpatient outcomes, and in supporting multidisciplinary coordination across medicine, nursing, respiratory therapy, nutrition services, case management, and palliative care teams.

From an implementation perspective, these models could be deployed as a risk flag within hospital analytics systems or electronic health record decision-support infrastructure using routinely coded administrative data, with periodic recalibration and local validation. To minimize unintended consequences, deployment should emphasize interpretability, audit for subgroup performance, and ensure that risk estimates are used to reduce missed deterioration and improve care coordination, not to restrict access to beneficial treatments. Prospective evaluation is needed to determine whether risk-guided workflows improve patient-centered outcomes, resource utilization, and equity in hospitalized patients with AD.

The models developed in this study are intended to support clinical decision-making rather than replace clinician judgment. In practice, the restricted model excluding do-not-resuscitate (DNR) orders and palliative care may be most applicable early during hospitalization, when end-of-life documentation is not yet available, to support admission-level risk stratification and prompt enhanced monitoring (e.g., early respiratory surveillance), timely nutritional assessment, and speech-language pathology evaluation when relevant. As hospitalization progresses, the full model incorporating DNR status and palliative care may better reflect recognized end-of-life trajectories and support goals-of-care discussions, multidisciplinary care coordination, and resource allocation.

This dual-model design is intended to align predictive tools with real-world workflows: the restricted model supports proactive risk identification early in the hospitalization, while the full model supports later-stage care planning once goals-of-care documentation is established.

### 4.6. Strengths and Limitations

This study’s strengths include the use of a large, nationally representative dataset, the integration of both regression and explainable AI frameworks, and a comprehensive evaluation of model performance and interpretability. The SHAP-based analysis addresses concerns about model opacity and offers clinically actionable insights through transparent feature contribution plots; however, SHAP is model-dependent and can be sensitive to correlated predictors, so they should be interpreted as explanatory rather than causal.

However, several limitations merit acknowledgment. First, the cross-sectional nature of the dataset limits causal inference, and observed associations may reflect severity at presentation rather than independent predictors. Second, variations in hospital coding practices, especially for palliative care, DNR, or dysphagia, may introduce misclassification bias. Third, our findings are based on 2017 data, and external validation across other years or patient populations is needed to assess temporal stability and generalizability. Lastly, hospital-level structural features such as bed capacity, staffing ratios, or care coordination processes were not included but may influence patient outcomes. Our analysis used the 2017 NIS (pre-COVID) because the pandemic substantially altered inpatient case-mix and care delivery; predictor profiles and model performance may differ in later years, and external validation using post-2020 NIS data is warranted. In addition, the NIS lacks direct measures of physiologic severity, functional status, dependence, mobility, and validated frailty indices, which are highly relevant to prognosis in AD. The dataset also does not capture prior institutionalization or long-term care residence, limiting differentiation between community-dwelling patients and nursing home residents. Finally, because this analysis reflects the U.S. healthcare context, caution is warranted in extrapolating findings to health systems with different organization, funding models, and long-term care availability.

These missing domains (physiologic severity, functional status/frailty, polypharmacy, and hospital operational factors) may limit model completeness by constraining the ability to capture baseline vulnerability and acute clinical trajectory with the granularity available in prospective clinical datasets. In addition, our analysis uses 2017 (pre-COVID-19) hospitalization data; inpatient case-mix, care pathways, and mortality patterns have changed after 2020. External validation and recalibration using more recent cohorts (e.g., 2020–2022 NIS) will be an important next step to assess temporal stability and post-pandemic generalizability.

Because in-hospital mortality was infrequent (4.7%), we avoided synthetic oversampling and instead emphasized evaluation metrics appropriate for imbalanced outcomes (including AUPRC and calibration) alongside AUROC. Future extensions could explore carefully validated approaches to data balancing and/or synthetic augmentation for rare outcomes, particularly when paired with rigorous calibration assessment and external validation. Recent work in rare-condition classification illustrates practical considerations for balancing strategies and generative approaches under class imbalance and may inform future methodological extensions in this domain [42].

### 4.7. Clinical and Policy Implications

Our findings offer several implications for frontline clinicians, hospital administrators, and health policy leaders. Clinically, the elevated SHAP importance of dysphagia, malnutrition, and aspiration suggests that earlier identification and intervention for feeding and swallowing impairments may mitigate downstream mortality risk. Additionally, identifying high-risk AD patients early, prior to palliative consultations or DNR documentation, can inform family discussions, care escalation decisions, and targeted supportive interventions.

From a policy perspective, the socioeconomic gradient observed across ZIP income quartiles highlights persistent disparities in outcomes, warranting equity-focused interventions in hospital-based dementia care. Furthermore, the successful application of explainable AI in this study serves as a proof-of-concept for broader implementation in predictive hospital analytics, particularly in frail older populations with multifactorial risk profiles. Future work could compare these models with a simple neural network baseline under the same hospital-grouped validation to quantify potential performance gains relative to reduced interpretability.

Future research should prioritize prospective validation incorporating functional status and frailty measures, evaluate subgroup-specific models (e.g., patients residing in long-term care settings or those with cardiometabolic multimorbidity), and conduct external validation across multiple years and healthcare systems to assess temporal stability and generalizability.

## 5. Conclusions

This study demonstrates the utility of combining regression and explainable AI to uncover mortality predictors in hospitalized AD patients. While end-of-life care markers like palliative consultations and DNR status dominate in magnitude, our results highlight the clinical relevance of acute organ dysfunction, swallowing impairment, and socioeconomic factors. The integration of SHAP-based insights enhances model transparency and supports nuanced interpretation of complex multimorbidity patterns. As healthcare systems increasingly adopt AI tools, our findings underscore the importance of balancing performance with interpretability, especially when caring for vulnerable, high-risk populations like those living with AD.

## Figures and Tables

**Table 1:** Cohort characteristics: NIS 2017 AD cohort aged ≥60 years.

Characteristic	Level	Weighted % or Mean (SE)	95% CI
Age, years	Mean (SE)	82.40 (0.04)	82.33–82.47
Sex	Male	38.3	37.9–38.6
	Female	61.7	61.4–62.1
Admission type	Non-elective	92.4	91.9–92.8
	Elective	7.6	7.2–8.1
Weekend admission	No	75.8	75.5–76.1
	Yes	24.2	23.9–24.5
In-hospital mortality	Died	4.7	4.5–4.8
Sepsis	Yes	15.7	15.4–16.1
Acute respiratory failure (ARF)	Yes	14.2	13.9–14.5
Acute kidney injury (AKI)	Yes	23.2	22.8–23.6
Aspiration	Yes	7.9	7.7–8.2
Urinary tract infection (UTI)	Yes	25.4	25.0–25.8
Malnutrition	Yes	8.2	7.9–8.5
Dysphagia	Yes	10.7	10.4–11.0
Pressure ulcer	Yes	7.2	6.9–7.4
Congestive heart failure (CHF)	Yes	23.0	22.7–23.4
Coronary artery disease (CAD)	Yes	25.7	25.3–26.1
Atrial fibrillation (AFib)	Yes	25.6	25.3–26.0
Cerebrovascular disease (CVA)	Yes	7.5	7.3–7.7
Anemia	Yes	12.7	12.4–13.0
Hypothyroidism	Yes	21.7	21.4–22.1
Do-Not-Resuscitate (DNR) order	Yes	32.1	31.4–32.7
Palliative care	Yes	11.3	10.9–11.6
Race	White	73.9	72.8–75.0
	Black	11.6	11.0–12.2
	Hispanic	9.3	8.4–10.2
	Asian or Pacific Islander	2.4	2.1–2.8
	Native American	0.3	0.22–0.37
	Other	2.5	2.17–2.83
ZIP income quartile	0–25th percentile (lowest income)	28.9	27.8–30.0
	26th–50th percentile	26.2	25.4–27.1
	51st–75th percentile	23.6	22.8–24.4
	76th–100th percentile (highest income)	21.3	20.2–22.4
Transfer-in (TRAN_IN)	Not transferred in	82.5	81.7–83.2
	Transferred in from a different acute care hospital	5.1	4.8–5.5
	Transferred in from another type of health facility	12.4	11.8–13.1
Hospital division	New England	4.8	4.3–5.4
	Middle Atlantic	13.5	12.7–14.4
	East North Central	16.5	15.5–17.5
	West North Central	6.8	6.2–7.5
	South Atlantic	20.9	20.0–22.0
	East South Central	7.9	7.1–8.7
	West South Central	12.5	11.8–13.3
	Mountain	4.1	3.8–4.5
	Pacific	12.9	12.1–13.7

**Table 2:** Survey-weighted logistic regression for in-hospital mortality (adjusted odds ratios).

Covariate	Category (Ref)	Adjusted OR	95% CI	*p*-Value
Age (years)	continuous	1.017	1.011–1.023	<0.001
Female	vs. Male	0.858	0.794–0.926	<0.001
Race (ref = White)	Black	1.050	0.924–1.193	0.455
	Hispanic	1.174	1.024–1.347	0.021
	Asian or Pacific Islander	1.079	0.866–1.344	0.497
	Native American	0.723	0.287–1.820	0.491
	Other	1.151	0.909–1.458	0.242
ZIP income quartile (ref = 0–25th percentile (lowest income))	26th–50th percentile	0.849	0.762–0.946	0.003
	51st–75th percentile	0.799	0.710–0.899	<0.001
	76th–100th percentile (highest income)	0.798	0.706–0.903	<0.001
Elective admission	vs. Non-elective	2.334	1.961–2.777	<0.001
Transfer-in (ref = Not transferred in)	Transferred in from a different acute care hospital	1.562	1.322–1.844	<0.001
	Transferred in from another type of health facility	1.124	1.004–1.257	0.042
Weekend admission	vs. Weekday	0.944	0.867–1.028	0.186
Hospital division (ref = New England)	Middle Atlantic	1.108	0.870–1.409	0.406
	East North Central	0.615	0.484–0.782	<0.001
	West North Central	0.757	0.580–0.989	0.041
	South Atlantic	0.701	0.557–0.883	0.003
	East South Central	1.064	0.793–1.427	0.680
	West South Central	0.788	0.618–1.007	0.056
	Mountain	0.569	0.421–0.769	<0.001
	Pacific	0.973	0.775–1.223	0.817
Sepsis	Yes vs. No	2.260	2.074–2.462	<0.001
Acute respiratory failure	Yes vs. No	5.148	4.730–5.602	<0.001
Acute kidney injury	Yes vs. No	1.466	1.349–1.592	<0.001
Aspiration	Yes vs. No	1.228	1.101–1.368	<0.001
Urinary tract infection	Yes vs. No	0.737	0.673–0.807	<0.001
Malnutrition	Yes vs. No	1.235	1.106–1.378	<0.001
Dysphagia	Yes vs. No	0.569	0.506–0.640	<0.001
Pressure ulcer	Yes vs. No	1.033	0.908–1.176	0.618
Congestive heart failure	Yes vs. No	1.074	0.981–1.175	0.124
Coronary artery disease	Yes vs. No	0.943	0.868–1.024	0.164
Atrial fibrillation	Yes vs. No	1.191	1.094–1.297	<0.001
Cerebrovascular disease	Yes vs. No	1.382	1.214–1.573	<0.001
Anemia	Yes vs. No	0.878	0.788–0.978	0.018
Hypothyroidism	Yes vs. No	0.941	0.860–1.031	0.192
Do-Not-Resuscitate (DNR) order	Yes vs. No	2.198	1.994–2.423	<0.001
Palliative care	Yes vs. No	6.189	5.589–6.853	<0.001

**Table 3:** Model Performance Comparison.

Model	Dataset Type	AUROC	AUPRC	Brier Score	Log Loss
XGBoost	Full Model	0.8866	0.3238	0.0364	0.1337
Logistic Regression	Full Model	0.8789	0.3103	0.0372	0.1375
XGBoost	Sensitivity (No DNR/Pall)	0.8106	0.2061	0.0403	0.1563
Logistic Regression	Sensitivity (No DNR/Pall)	0.8059	0.2056	0.0403	0.1569

Note: DNR: Do-Not-Resuscitate; Pall: Palliative Care.

**Table 4:** Top Predictors of In-Hospital Mortality Among AD Patients.

Rank	Predictor	Logistic Coefficient	XGBoost Gain
1	Palliative Care	4.554	14.703
2	Acute Respiratory Failure	2.466	11.423
3	Acute Kidney Injury	1.437	4.545
4	Dysphagia	−1.301	4.358
5	Age	1.273	4.909
6	Aspiration Pneumonia	0.950	4.257
7	Urinary Tract Infection	−0.842	3.697
8	Elective Admission	−0.734	1.059
9	Pressure Ulcers	−0.724	3.320
10	Stroke	−0.672	3.023
11	Sepsis	0.663	3.777
12	Anemia	0.637	2.765
13	Congestive Heart Failure	0.535	2.711
14	Malnutrition	0.326	3.579
15	Coronary Artery Disease	0.290	3.343

## Data Availability

The data used in this study are available from the Agency for Healthcare Research and Quality (AHRQ) Healthcare Cost and Utilization Project (HCUP) Nationwide Inpatient Sample (NIS) (https://www.hcup-us.ahrq.gov/, accessed on 1 January 2026) under a data use agreement and are not publicly shareable by the authors.

## Supplementary Materials

The following supporting information can be downloaded at: https://www.mdpi.com/article/10.3390/geriatrics11020023/s1, Table S1: ICD-10-CM Codes for Comorbidities Used in the Study.

## Abbreviations

The following abbreviations are used in this manuscript:

AD	Alzheimer’s disease
AI	artificial intelligence
AKI	acute kidney injury
aOR	adjusted odds ratio
AUPRC	area under the precision–recall curve
AUROC	area under the receiver operating characteristic curve
ARF	acute respiratory failure
DNR	do-not-resuscitate
HCUP	Healthcare Cost and Utilization Project
ICD-10-CM	International Classification of Diseases, 10th Revision, Clinical Modification
NIS	Nationwide Inpatient Sample
SHAP	SHapley Additive exPlanations
XGBoost	eXtreme Gradient Boosting
