# Supplementary material for: Risk Stratification for In-Hospital Mortality in Alzheimer’s Disease Using Interpretable Regression and Explainable AI
Source: Geriatrics (Basel). 2026 Feb 24;11(2):23. doi: 10.3390/geriatrics11020023 (PMC13010599; doi:10.3390/geriatrics11020023)
Supplement: Supplementary file 1 [file geriatrics-11-00023-s001.zip › geriatrics-4104741-supplementary.pdf]

Table S1. ICD-10-CM Codes for Comorbidities Used in the Study.

Comorbidity	ICD-10-CM Code(s) or Prefixes	Description / Notes
Sepsis	A40*, A41*	Septicemia, sepsis
Acute Respiratory Failure	J96.0*, J96.2*	Acute respiratory failure with or without hypoxia
Acute Kidney Injury	N17*	Acute renal failure
Aspiration	J69.0*	Aspiration pneumonia
Urinary Tract Infection	N39.0	UTI, site not specified
Malnutrition	E40–E46*	Protein-energy malnutrition
Dysphagia	R13*	Difficulty swallowing
Pressure Ulcer	L89*	Pressure injuries
Congestive Heart Failure	I50.2*, I50.3*	CHF with systolic/diastolic dysfunction
Coronary Artery Disease	I25.1, I25.7, I25.9	Atherosclerotic heart disease
Atrial Fibrillation	I48*	Atrial fibrillation and flutter
Cerebrovascular Disease	I63*, I69.3*	Ischemic stroke and its sequelae
Anemia	D50–D64*	Nutritional, aplastic, and other anemias
Hypothyroidism	E03*	Other hypothyroidism
Do-Not-Resuscitate	Z66	Presence of DNR order
Palliative Care	Z51.5	Encounter for palliative care
